# First data on bacteria associated with bat ectoparasites collected in Kharkiv oblast, Northeastern Ukraine

**DOI:** 10.1186/s13071-022-05582-3

**Published:** 2022-11-25

**Authors:** Anton Vlaschenko, Cristian Răileanu, Oliver Tauchmann, Denys Muzyka, Valeria Bohodist, Serhii Filatov, Olena Rodenko, Ihor Tovstukha, Cornelia Silaghi

**Affiliations:** 1 LLC “ASTRAVIR TECHNOLOGY”, Poltavskyi Shliakh, 6, 25, Kharkiv, 61001 Ukraine; 2 Bat Rehabilitation Center of Feldman Ecopark, Lesnoye, Kharkiv Region, 62340 Ukraine; 3 Institute of Natural Sciences, Department of Zoology, H.S. Skovoroda Kharkiv National Pedagogical University, Valentynivska St., 2, Kharkiv, 61168 Ukraine; 4 NGO “Ukrainian Independent Ecology Institute”, Plekhanov St., 40, Kharkiv, 61001 Ukraine; 5 Institute of Infectology, Friedrich-Loeffler-Institut, Suedufer 10, 17493 Greifswald-Isle of Riems, Germany; 6 National Scientific Center “Institute of Experimental and Clinical Veterinary Medicine”, Pushkinska St., 83, Kharkiv, 61023 Ukraine; 7 Veterinary Medicine Department, Bila Tserkva National Agrarian University, Stavishchanskaya St., 126, Bila Tserkva, 09111 Ukraine; 8 Department of Pediatrics and the National School of Tropical Medicine, Baylor College of Medicine, Houston, TX USA; 9 Kharkiv International Medical University, Molochna St., 38, Kharkiv, 61001 Ukraine; 10 Department of Biology, University of Greifswald, Domstraße 11, 17489 Greifswald, Germany

**Keywords:** Chiroptera, Microbiota, Ectoparasites, Zoonoses, vector-borne, Ukraine

## Abstract

**Background:**

Bats (*Mammalia*: *Chiroptera*) serve as natural reservoirs for many zoonotic pathogens worldwide, including vector-borne pathogens. However, bat-associated parasitic arthropods and their microbiota are thus far not thoroughly described in many regions across the globe, nor is their role in the spillover of pathogens to other vertebrate species well understood. Basic epidemiological research is needed to disentangle the complex ecological interactions among bats, their specific ectoparasites and microorganisms they harbor. Some countries, such as Ukraine, are particularly data-deficient in this respect as the ectoparasitic fauna is poorly documented there and has never been screened for the presence of medically important microorganisms. Therefore, the aims of this study were to provide first data on this topic.

**Methods:**

A total of 239 arthropod specimens were collected from bats. They belonged to several major groups of external parasites, including soft ticks, fleas, and nycteribiid flies from six chiropteran species in Northeastern Ukraine. The ectoparasites were individually screened for the presence of DNA of *Rickettsia* spp., *Anaplasma*/*Ehrlichia* spp., *Bartonella* spp., *Borrelia* spp., and *Babesia* spp. with conventional PCRs. Positive samples were amplified at several loci, sequenced for species identification, and subjected to phylogenetic analysis.

**Results:**

*Rickettsia* DNA was detected exclusively in specimens of the soft tick, *Carios vespertilionis* (7 out of 43 or 16.3%). Sequencing and phylogenetic analysis revealed high similarity to sequences from *Rickettsia parkeri* and several other *Rickettsia* species. Bacteria from the family *Anaplasmataceae* were detected in all groups of the ectoparasites (51%, 122/239 samples), belonging to the genera *Anaplasma*, *Ehrlichia*, and *Wolbachia*. The detection of *Bartonella* spp. was successful only in fleas (*Nycteridopsylla eusarca*) and bat flies *(Nycteribia koleantii, N. pedicularia*), representing 12.1% (29/239) of the collected ectoparasites. No DNA of *Babesia* or *Borrelia* species was identified in the samples.

**Conclusions:**

We report for the first time in Ukraine the molecular detection of several bacterial agents in bat ectoparasites collected from six species of bats. The data presented extend the knowledge on the distribution of ectoparasite species in bats and their involvement in potentially circulating agents pathogenic for humans and vertebrate animals.

**Graphical Abstract:**

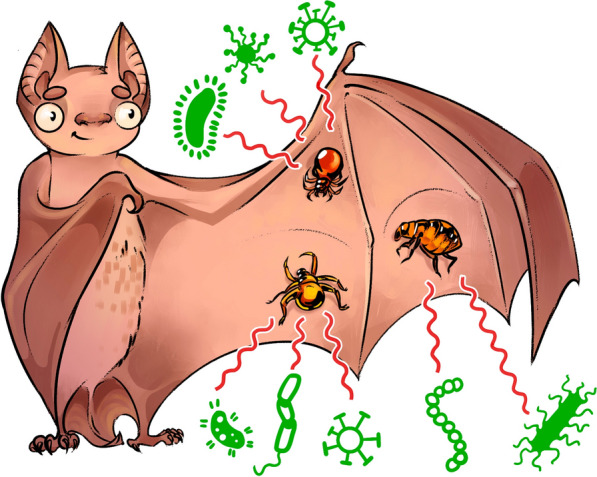

**Supplementary Information:**

The online version contains supplementary material available at 10.1186/s13071-022-05582-3.

Bats (*Mammalia*: *Chiroptera*) represent the second-most diverse order of mammals after rodents [1]. The multitude of their ecological interactions with other animals and their shared physical environment puts bats in close contact with a large variety of viruses, bacteria, fungi, and parasites [2, 3]. Currently, bat microbiota are poorly documented and understood although during the last 2 decades bats received increased research attention as a natural source of well-known and potentially zoonotic pathogens, especially viruses [4]. While the role of bats in circulation and spillover of zoonotic viruses such as lyssaviruses, filoviruses, henipaviruses, and coronaviruses is relatively well established [5, 6], limited knowledge exists regarding their role as reservoirs for arthropod-borne pathogens, which represent a substantial proportion of zoonoses worldwide [7].

Previous research mostly focused on detection of vector-borne bacteria from the genera *Bartonella, Rickettsia*, and *Borrelia* in samples of bat tissues, excreta, and their ectoparasites [8–12]. More research efforts are needed to determine the relevance of these findings to the circulation of zoonotic vector-borne pathogens or their significance for human and animal health.

Many areas, especially in Eastern Europe, are thus far lacking this type of research. For example, very little is known about bat ectoparasites in Ukraine, and the vector-borne bacteria they might carry have never been surveyed in the country [13–15]. The Kharkiv oblast (synonym with region), Northeastern Ukraine, is the most intensively studied region in the country, with 20 years of bat-related stationary research and monitoring activities, where the bat diversity reaches 15–16 species [16]. However, bat ectoparasites have thus far not been the main research focus, and there is only one publication dedicated to bat ectoparasites in the region, which was published in a local journal [15]. The authors identified eight species of ectoparasites, including mesostigmatid mites (genera *Spinturnix*: 3 spp.; *Macronyssus*: 1 sp.; *Steatonyssus*: 1 sp.), fleas (genus *Ischnopsyllus*: 2 spp.), and a bat fly (*Nycteribia koleantii*) occurring in five bat taxa. Moreover, they examined only a small number of opportunistically collected samples (total of 142 specimens), and the ectoparasites were not screened for microorganisms [15]. Thus, our present study is aimed at filling the gap by conducting molecular screening of selected vector-borne microorganisms in recent samples of ectoparasitic arthropods collected from bats in Kharkiv oblast.

The field survey was conducted in five localities divided among the three habitat types: urban (November 2019), natural (August–September 2019), and rural (July 2020), representing main types of land use in the region (Additional file 1: Table S1). Bats were mist-netted in autumn swarming and wintering sites or hand captured from two breeding colonies that roosted in private houses in a countryside area. Each individual was identified to the species level, its sex, age, and reproductive status were noted, and forearm length and body mass were measured (for details see: [17]). After taking the measurements from a bat, the body, coat, ears, wing, and uropatagium membranes of each animal were examined for ectoparasites in daylight or using a headlamp. All detected arthropods were collected with tweezers and cotton swabs and placed in individually labeled tubes with 96% ethanol. From the total number of collected ectoparasites (~ 1000), so far 239 specimens have been morphologically identified to the species level using the Nikon SMZ800 stereomicroscope and taxonomic keys [18–20]. After the morphological identification, these specimens were transferred to 70% ethanol and sent to the Institute of Infectology, Friedrich-Loeffler-Institut, Germany, for further molecular screening.

The samples were processed individually, each ectoparasite being homogenized in sterile phosphate-buffered saline (PBS) with steel beads using the Tissue Lyser II (Qiagen, Hilden, Germany). The DNA extraction was performed from 100 μl aliquots using NucleoMag^®^ VET kit (Macherey–Nagel, Düren, Germany) and the King Fisher^®^ Flex Purification system (ThermoFisher, Darmstadt, Germany), according to the manufacturer’s instructions. Total DNA was eluted in 100 μl elution buffer and then stored at −80 °C until further analysis.

Four species of ectoparasites collected from different bat species (Table 1) were PCR screened for the presence of *Babesia* spp., *Rickettsia* spp., *Bartonella* spp., and *Anaplasma/Ehrlichia* spp., while only *Carios vespertilionis* ticks were additionally screened for DNA of *Borrelia* spp. The screening was done using specific primers listed in Additional file 1: Table S2 and PCR conditions described in the publications cited therein. The reaction products from samples with successfully amplified target genes were purified with NucleoSEQ^®^ kit (Mackerey Nagel, Düren, Germany) following manufacturer’s instructions and Sanger sequenced in the Laboratory for Applied Bioinformatics and Sequencing of Viral Genomes and Transcriptomes, Institute of Diagnostic Virology, Friedrich-Loeffler-Institut.

**Table 1:** Ectoparasites collected from bats in Kharkiv oblast, NE Ukraine, that were includes in the analysis

Bat host species	*Carios vespertilionis*^a^	*Nycteridopsylla eusarca*	*Nycteribia kolenatii*	*Nycteribia pedicularia*	Total
*Myotis dasycneme*	28	0	0	0	28
*Myotis daubentonii*	2	0	78	18	98
*Nyctalus noctula*	0	100	0	0	100
*Pipistrellus kuhlii*	1	0	0	0	1
*Pipistrellus pygmaeus*	11	0	0	0	11
*Plecotus auritus*	1	0	0	0	1
Total	43	100	78	18	239

^a^Blood-fed larvae

Among the tested samples, DNA of *Rickettsia* spp. was identified in 2.9% (7/239) of ectoparasites (Table 2). All positive samples were *C. vespertilionis* ticks, six specimens collected from *Pipistrellus pygmaeus* and one from *P. kuhlii* bat species. Sequence analysis of the rickettsial *gltA* gene did not successfully differentiate the species, with all detected sequences showing 100% similarity to several *Rickettsia* spp.: *Rickettsia parkeri* (GenBank access. no.: MK814825), *R. africae* (MH938655), *R. sibirica* (KU310587), or uncultured *Rickettsia* sp. (MG228263). PCR based on the *ompA* gene followed by sequencing of positive *C. vespertilionis* ticks for *Rickettsia* indicated 100% similarity of all samples with *R. parkeri* (MK962698) and *Rickettsia* sp. (KX137902).

**Table 2:** Overall results of molecular screening of ectoparasites collected from bats in Kharkiv oblast, NE Ukraine

Screened agents	*Carios vespertilionis*	*Nycteridopsylla eusarca*	*Nycteribia kolenatii*	*Nycteribia pedicularia*	Total
	[%, (n)]	[%, (n)]	[%, (n)]	[%, (n)]	[%, (n)]
*Rickettsia* spp.	16.3% (7/43)	0	0	0	2.9% (7/239)
*Bartonella* spp.	0	7%** (7/100)	21.8%^ns^ (17/78)	27.8%^ns^ (5/18)	12.1% (29/239)
*Anaplasma/Ehrlichia* spp.	4.7%*** (2/43)	56% ^ns^ (56/100)	69.2% ^ns^ (54/78)	55.6% ^ns^ (10/18)	51% (122/239)
*Babesia* spp.	0	0	0	0	0
*Borrelia* spp.	0	n.a	n.a	n.a	0

%: percentage of positive samples; *n*: number of positive samples out of the total tested; –: *n.a.*, not applicable

Statistical analysis: ANOVA, Tukey’s test, *P* < 0.05: ***significant, *P* < 0.001; **significant, *P* = 0.014; ^ns^: non-significant

The BLASTn analysis for the *Rickettsia* ompB sequences also showed 100% similarity of the samples from this study with *R. parkeri* (CP040325), uncultured *Rickettsia* sp. (MK405417), and *Rickettsia* sp. (AF123720).

The phylogenetic analysis (Mega X [21]) was done using the *gltA*, *ompA*, and *ompB* concatenated *Rickettsia* sequences and sequences from representative *Rickettsia* species available in GenBank. The analysis demonstrates that all *Rickettsia* sequences detected in *C. vespertilionis* in this study are phylogenetically closely related to *R. parkeri* and *R. africae* (Fig. 1a).

**Fig. 1:**
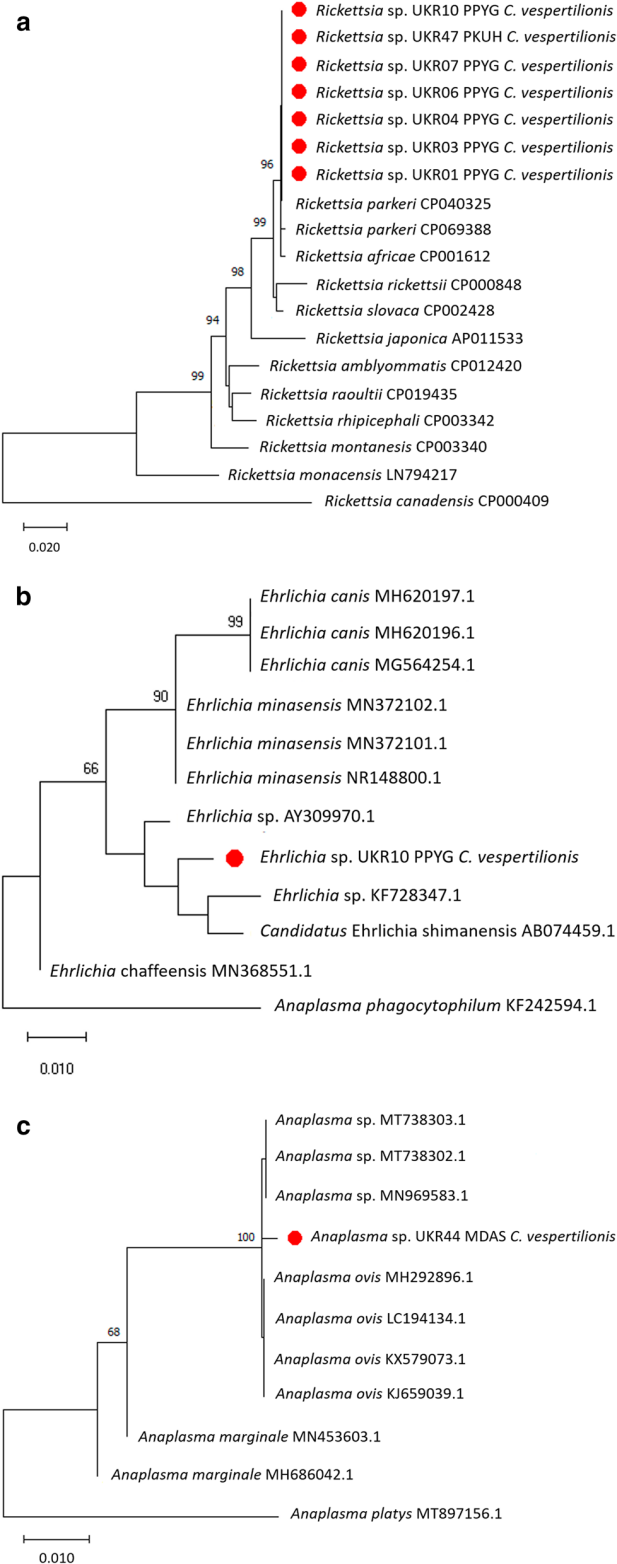
Phylogenetic analysis of sequences within the Rickettsiales order. **a** Phylogenetic tree of *Rickettsia* sequences based on concatenated *gltA*, *ompA*, and *ompB* genes using the maximum likelihood analysis and Tamura three parameter with discrete gamma distribution; **b** phylogenetic analysis of *Ehrlichia* detected in *C. vespertilionis* based on the *16S rRNA* loci, using the maximum likelihood analysis and Hasegawa-Kishino-Yano; **c** phylogenetic analysis of *Anaplasma* detected in *C. vespertilionis* based on the *16S rRNA* loci, using the maximum likelihood analysis and Tamura three parameter. Bootstrap values are indicated at the nodes. The red dot preceding the sample names indicates the sequences obtained in this study

The detection of *R. parkeri*-like sequences in bat-associated soft ticks from Europe represents a noteworthy finding as this alphaproteobacterium is known to be associated primarily with hard ticks in the genus *Amblyomma* occurring in the Americas [22]. However, a recent study ostensibly identified *R. parkeri* sequences in tissues of *Pipistrellus pipistrellus* bats from China [23], which would significantly expand the geography and host range of the species. While some of the strains in *R. parkeri* sensu lato complex are well-established human pathogens, little is known about their natural reservoir hosts [22].

Moreover, neither pathogenicity in vertebrates nor transmissibility by argasid ticks is known for the *R. parkeri*-like species presently detected in Eurasia. Further research efforts, therefore, should focus on isolating and establishing the identity of the bacterium as well as elucidating its enzootic cycle. This would also be of public health relevance, keeping in mind that *C. vespertilionis* may occasionally bite humans in situations when their bat hosts are no longer available [24].

Regarding DNA of *Anaplasma/Ehrlichia* spp., 51% (122/239) of the samples tested positive: 4.7% (2/43) of *C. vespertilionis*, 56% (56/100) of *Nycteridopsylla eusarca*, 69.2% (54/78) of *Nycteribia kolenatii*, and 55.6% (10/18) of *N. pedicularia* (Table 2). The two *C. vespertilionis* samples positive for *Anaplasma*/*Ehrlichia* spp. after the initial *16S* rRNA PCR were further amplified by hemi-nested PCR targeting a *16S* rRNA fragment then sequenced. The DNA sequence analysis showed that one sequence was 98.5% similar to *Candidatus* *Ehrlichia shimanensis* (AB074459) while the other sample had 99.7% similarity to uncultured *Anaplasma* sp. clone Erz1600 (MT601947). The phylogenetic analysis based on the *16S* rRNA partial sequence indicates that the *Ehrlichia* sequence detected in *C. vespertilionis* clusters in a clade that includes *Candidatus* Ehrlichia shimanensis, uncultured *Ehrlichia*: *E. minasensis* or *E. canis* (Fig. 1b)*.* The phylogenetic tree based on the *16S* rRNA partial sequence shows that the *Anaplasma* sequence was detected in *C. vespertilionis* clusters in a clade that includes uncultured *Anaplasma* and *Anaplasma ovis* (Fig. 1c)*.* Eight *N. eusarca* samples positive for *Anaplasma/Ehrlichia* spp. were sequenced, two having 100% identity to uncultured bacterium clone layman_j06 (DQ980970), and six sequences were 99.6–100% similar to *Wolbachia* endosymbiont (MH618381). Four *N. pedicularia* and six *N. kolenatii* flies positive for *Anaplasma/Ehlichia* spp. were also sequenced, having 99.3–100% similarity to *Wolbachia* endosymbiont (MH618380).

While zoonotic *Anaplasmataceae* such as *Anaplasma phagocytophilum* have been previously reported from insectivorous bats and their ticks in Europe [12], it remains unclear whether bats play any role in the epidemiology of granulocytic anaplasmosis. Most of the hits in the genera *Ehrlichia* and *Anaplasma* from the present study are clustering with poorly characterized species (e.g. *E. minasensis*) or with microorganisms not commonly associated with bats, such as *E. canis* or *A. ovis*. This implies that either a much broader range of vertebrate reservoirs and arthropod vectors support circulation of these pathogens in nature or, more parsimoniously, that the molecular markers selected for species identification have poor discriminatory capacity at this level. On the other hand, the finding of such endosymbionts as *Wolbachia* sp. is not surprising as these bacteria are almost universally present in many groups of arthropods and in filarial nematodes and have been reported previously in multiple bat fly species [25].

The detection of *Bartonella* spp. was successful in 12.1% (29/239) ectoparasites collected from bats. All bat ticks tested negative, while *N. eusarca, N. kolenatii*, and *N. pedicularia* showed prevalence rates that varied from 7 to 21.8% and 27.8%, respectively (Table 2). The analysis based on the *gltA* gene of DNA sequences from *N. eusarca* revealed 99–100% similarity to uncultured *Bartonella* sp. clone 198T155 (MK140218) detected in *C. vespertilionis* from The Netherlands (*n* = 3 isolates) and uncultured *Bartonella* sp. isolate M451 (AJ871615) found in the blood of *Nyctalus noctula* from UK (*n* = 4 isolates). Eight *N. kolenatii*- and two *N. pedicularia*-positive samples for *Bartonella 16S-23S* rRNA were also sequenced. Sequence analysis indicated that three sequences from *N. kolenatii* had 94.5% similarity to *Bartonella* sp. strain 44601 (MF288119) obtained from *Myotis blythii*, and two sequences matched 98.7% and 99%, respectively, to *Bartonella* sp. strain 44718 (MF288128) from *Pipistrellus pygmaeus*. The other five sequences (three from *N. kolenatii* and two from *N. pedicularia*) had 96.7–97.4% similarity to uncultured *Bartonella* sp. clone 137 (KX420735) found in *Rhinolophus ferrumequinum* bat species.

*Bartonella gltA* and *16S-23S* rRNA sequences were further used to construct phylogenetic trees (Fig. 2a, b). The phylogenetic analysis based on the *gltA* partial sequence shows that three *Bartonella* sequences detected in *N. eusarca* cluster in a clade that includes uncultured *Bartonella* (MK140218) and *Bartonella washoensis* (AF050108). The additional four obtained sequences cluster in a separate clade. The analysis based on the partial *16S-23S* rRNA sequence shows that *Bartonella* sequences detected in *N. kolenatii* and *N. pedicularia* cluster close to uncultured *Bartonella* (KX420735) and *Bartonella* sp. (MF288119 and MF288128). All sequence data and accession nos. are shown in Additional file 1: Table S3.

**Fig. 2:**
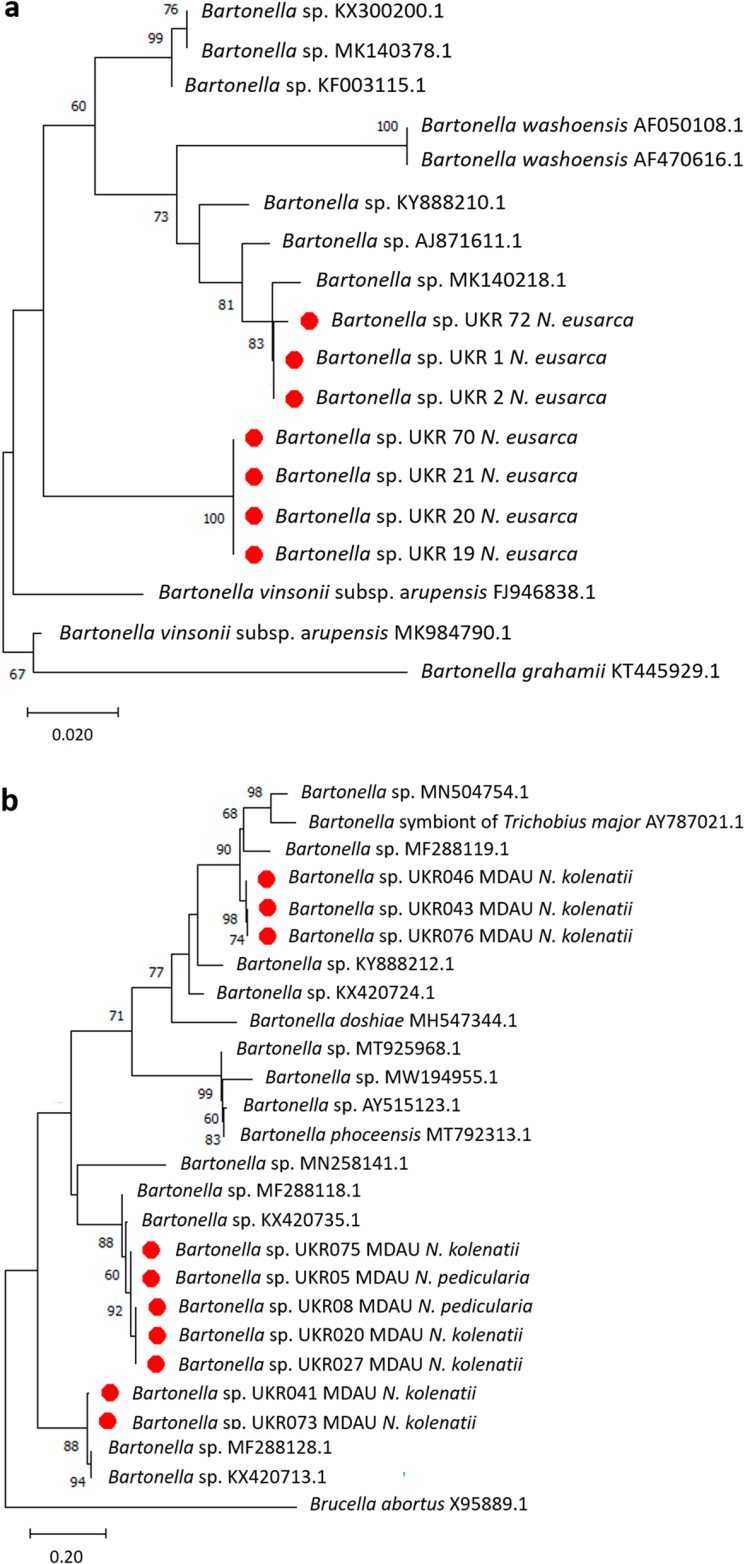
Phylogenetic analysis of *Bartonella* spp. isolates obtained in this study. **a** Phylogenetic tree based on citrate synthase (*gltA*) partial gene using the maximum likelihood analysis and Tamura three-parameter model with a discrete gamma distribution; **b** phylogenetic analysis of *Bartonella* spp. based on 16S-23S rRNA sequence using the maximum likelihood analysis and Tamura three parameter with a discrete gamma distribution. Bootstrap values are indicated at the nodes. The red dots highlight the sequences of this study. *Brucella abortus* (X95889) was used as outgroup

Various *Bartonella* sequences have been detected in many bat species and in their ectoparasites (fleas, ticks, and flies) across the world [12, 26]. While some of those findings are thought to represent zoonotic bacteria [27], others cluster with sequences detected only in bats, bats and Nycteribiidae flies, or solely in bat flies, with no known vertebrate host association [28, 29]. It has been hypothesized that pathogenic bartonellae evolved from insect-specific ancestors through their association with hematophagous vectors, which allowed them to adapt to mammalian blood [30]. While currently pathogenic *Bartonella* spp. are believed to be highly host/vector specific, the remarkable diversity of sequences belonging to this genus found in bats and their ectoparasites suggests the ancient nature and evolutionary importance of this association [31].

All ectoparasite samples were negative for *Babesia* spp., while only *C. vespertilionis* were screened for *Borrelia* spp. and were positive in 4.7% (2/43) using the *16S-23S* IGS specific primers [32]. The following sequencing attempts for the locus were unsuccessful, suggesting non-specific amplification and leaving the samples without further identification.

This study offers first glimpses on the microbial diversity found in ectoparasites collected from several species of insectivorous bats in Northeast Ukraine. Our research effort creates the impetus for disentangling the vector-host-pathogen interactions among bats and their ectoparasites in an understudied part of Europe. Further studies employing larger sample sizes, greater diversity of the host and parasite species, and variable methods, including next generation sequencing, should reveal a complete and more complex picture. Given the globally changing patterns of bat distribution, their increasing proximity to humans, and the high rates of the infectious disease emergence in wildlife, domestic animals, and human populations, this basic research is important from a public health perspective as well as for conservation biology.

## Supplementary Information

**Additional file 1: Table S1**. Locations and roosting sites of bats collected for the purpose of this study. **Table S2**. Primers used for pathogen screening of ectoparasites. **Table S3. **GenBank accession numbers for sequences obtained in this study.

## Data Availability

The datasets supporting the conclusions of this article are included within the article and its additional files. Newly generated sequences used for the phylogenetic analysis were submitted to the GenBank database. All sequence data and accession nos. are shown in Table S3.
