# Supplementary material for: First data on bacteria associated with bat ectoparasites collected in Kharkiv oblast, Northeastern Ukraine
Source: Parasit Vectors. 2022 Nov 25;15:443. doi: 10.1186/s13071-022-05582-3 (PMC9700949; doi:10.1186/s13071-022-05582-3)
Supplement: Supplementary file 1 — Additional file 1: Table S1. Locations and roosting sites of bats collected for the purpose of this study. Table S2. Primers used for pathogen screening of ectoparasites. Table S3. GenBank accession numbers for sequences obtained in this study. [file 13071_2022_5582_MOESM1_ESM.docx]

**First data on bacteria associated with bat ectoparasites collected in Kharkiv oblast, Northeastern Ukraine**

Anton Vlaschenko, Cristian Răileanu, Oliver Tauchmann, Denys Muzyka, Valeria Bohodist, Serhii Filatov, Olena Rodenko, Ihor Tovstukha, Cornelia Silaghi

**Supplementary Table S1**. Locations and roosting-sites of bats collected for the purpose of this study

| **Habitat** | **Location and method of bat collecting** | **Season and period of collection** | **Species of inspected bats and number of individuals (n)** |
| --- | --- | --- | --- |
| Urban | Kharkiv city,  (50°00’21’’N 36°13’45”E)  Hibernation roost in a multi-storey building.  Handling in a roosting site. | Hibernation,  November 2019 | ***Nyctalus noctula***  (13 ind.) (*Nycteridopsylla eusarca* 100 ind.) |
| Natural | Liptsy mines,  (50°12’28”N 36°25’02”E)  Mass bat winter aggregation.  Mist-netting near entrances. | Swarming,  August 2019 | ***Myotis daubentonii*** (56 ind.),(*Nycteribia kolenatii* 78 ind*., Nycteribia pedicularia* 18 ind.*)*  ***M. dasycneme*** (2 ind.), (*Carios vespertilionis* 27 ind.)  ***Plecotus auritus*** (1 ind.) (*Carios vespertilionis* 1 ind.) |
|  | Open pit, Zavody vil.,  (49°09’15”N 37°04’04”E)  Mass bat autumn-swarming location.  Mist-netting near entrances. | Swarming,  September 2019 | ***M. daubentonii*** (1 ind.), (*Carios vespertilionis* 2 ind.)  ***M. dasycneme*** (1 ind.) (*Carios vespertilionis* 1 ind.*)* |
| Rural | Rosinka, Bugayevka vil.,  (50°08’40”N 36°52’05”E)  Roosting-site in a private house.  Handling in a roosting site. | Breeding,  July 2020 | ***Pipistrellus pygmaeus*** (8 ind.)  (*Carios vespertilionis* 11 ind.) |
|  | Slatino vil.  (50°12’33”N 36°09’32”E)  Roosting-site in a private house.  Handling in a roosting site. |  | ***P. kuhlii*** (1 ind.)  (*Carios vespertilionis* 1 ind.) |

Bat host species are in bold

**Supplementary Table S2**. Primers used for pathogen screening of ectoparasites

| **Target Gene** | **Reaction** | **Sequence (5’-3’)** | **Amplicon size (bp)** | **Annealing** | **Reference** |
| --- | --- | --- | --- | --- | --- |
| ***Rickettsia* spp.** | | | | | |
| *gltA*^1)^ | PCR | Rsfg877: GGGGGCCTGCTCACGGCGG* Rfsg1258: ATTGCAAAAAGTACAGTGAACA* | 381 | 56°C | [1] |
| *ompA*^1)^ | PCR | Rr190.70p: ATGGCGAATATTTCTCCAAAA* Rr190.701n: GTTCCGTTAATGGCAGCATCT* | 631 | 46°C | [2] |
| *ompB*^2)^ | Nested PCR | PCR 1:  ompB-OF: GTAACCGGAAGTAATCGTTTCGTAA  ompB-OR: CTTTATAACCAGCTAAACCACC  Nested PCR:  ompB SFG-IF: GTTTAATACGTGCTGCTAACCAA*  ompB SFG/TG-IR: GGTTTGGCCCATATACCATAAG* | 489  425 | 54°C  56°C | [3] |
| 16S rRNA^3)^ | Hemi-nested PCR | PCR 1:  16S8F: AGAGTTTGATCCTGGCTCAG  16S1507R: GTGAAGTCGTAACAAGGTA  Hemi-nested PCR:  16S8F: AGAGTTTGATCCTGGCTCAG  R16SR1: GGTGGTYGCGGATCGCAGAG |  | 56°C  56°C | [4] |
| ***Bartonella* spp.** | | | | | |
| *gltA*^1)^ | PCR | Bart781: GGGGACCAGCTCATGGTGG  Bart1137: AATGCAAAAAGAACAGTAAACA* | 380-400 | 52°C | [5] |
| 16s-23S rRNA^2)^ | PCR | BA325s: CTTCAGATGATGATCCCAAGCCTTCTGGCG  BA1100as: GAACCGACGACCCCCTGCTTGCAAAGCA* | 420-780 | 66°C | [6] |
| ***Anaplasma/Ehrlichia* spp.** | | | | | |
| 16S rRNA | PCR | EHR16SD: GGTACCYACAGAAGAAGTCC  EHR16SR: TAGCACTCATCGTTTACAGC* | 345 | 53°C | [7] |
| 16S rRNA^3)^ | Hemi-nested PCR | PCR 1:  fD1: AGAGTTTGATCCTGGCTCAG  EHR16SR: TAGCACTCATCGTTTACAGC  Hemi-nested PCR:  fD1: AGAGTTTGATCCTGGCTCAG  GA1UR: GAGTTTGCCGGGACTTCTTCT* | 760  426 | 55°C  56°C | [8] |
| ***Babesia/Theileria* spp.** | | | | | |
| 18S rRNA | PCR | BJ1: GTCTTGTAATTGGAATGATGG BN2: TAGTTTATGGTTAGGACTACG | 411-452 | 55°C | [9] |
| ***Borrelia* spp.** | | | | | |
| 16S-23S^3)^ rRNA | Nested PCR | PCR 1:  Bospp-IGS-F: GTATGTTTAGTGAGGGGGGTG  Bospp-IGS-R: GGATCATAGCTCAGGTGGTTAG  Nested PCR:  Bospp-IGS-Fi: AGGGGGGTGAAGTCGTAACAAG  Bospp-IGS-Ri: GTCTGATAAACCTGAGGTCGGA | 1007  388-685 | 56°C  58°C | [10] |

1), Target used for the analysis of *Carios vespertilionis* and *Nycteridopsylla eusarca* samples only; 2), Gene target used for the analysis of *C. vespertilionis*, *N. kolenatii* and *N. pedicularia*; 3), Only *C. vespertilionis* ticks were tested; *, Primers used in sequencing PCR.

**Supplementary Table S3.** GenBank accession numbers for sequences obtained in this study

| **Organism** | **Sample ID** | **Target Gene** | **GenBank access. no.** |
| --- | --- | --- | --- |
| *Rickettsia* spp. | UKR_gltA_C._vespertilionis* | *gltA* | ON260921 |
| *Rickettsia* spp. | UKR_ompA_C._vespertilionis* | *ompA* | ON260922 |
| *Rickettsia* spp. | UKR_ompB_C._vespertilionis* | *ompB* | ON260923 |
| *Bartonella* spp. | UKR_72_N._eusarca | *gltA* | ON511313 |
| *Bartonella* spp. | UKR_70_N._eusarca | *gltA* | ON511312 |
| *Anaplasma* spp. | UKR_44_MDAS_C._vespertilionis | *16S rRNA* | ON455120 |
| *Bartonella* spp. | UKR_21_N._eusarca | *gltA* | ON511311 |
| *Bartonella* spp. | UKR_20_N._eusarca | *gltA* | ON511310 |
| *Bartonella* spp. | UKR_19_N._eusarca | *gltA* | ON511309 |
| *Bartonella* spp. | UKR_2_N._eusarca | *gltA* | ON511308 |
| *Bartonella* spp. | UKR_1_N._eusarca | *gltA* | ON511307 |
| *Bartonella* spp. | UKR076_MDAU_N._kolenatii | *16S-23S rRNA* | ON477949 |
| *Bartonella* spp. | UKR075_MDAU_N._kolenatii | *16S-23S rRNA* | ON477950 |
| *Bartonella* spp. | UKR073_MDAU_N._kolenatii | *16S-23S rRNA* | ON477951 |
| *Bartonella* spp. | UKR046_MDAU_N._kolenatii | *16S-23S rRNA* | ON477952 |
| *Bartonella* spp. | UKR043_MDAU_N._kolenatii | *16S-23S rRNA* | ON477953 |
| *Bartonella* spp. | UKR041_MDAU_N._kolenatii | *16S-23S rRNA* | ON477954 |
| *Bartonella* spp. | UKR027_MDAU_N._kolenatii | *16S-23S rRNA* | ON477955 |
| *Bartonella* spp. | UKR020_MDAU_N._kolenatii | *16S-23S rRNA* | ON477956 |
| *Ehrlichia* spp. | UKR10_PPYG_C._vespertilionis | *16S rRNA* | ON455121 |
| *Bartonella* spp. | UKR08_MDAU_N._pedicularia | *16S-23S rRNA* | ON477957 |
| *Bartonella* spp. | UKR05_MDAU_N._pedicularia | *16S-23S rRNA* | ON477958 |

**Rickettsia* gltA, ompA and ompB sequences were submitted each as one operational taxonomic unit (isolates showed for each target gene 100% homology between them).

**References**

1. Regnery RL, Spruill CL, Plikaytis BD. Genotypic Identification of *Rickettsiae* and Estimation of Intraspecies Sequence Divergence for Portions of 2 Rickettsial Genes. J Bacteriol. 1991;173 5:1576-89; doi: DOI 10.1128/jb.173.5.1576-1589.1991.

2. Roux V, Fournier PE, Raoult D. Differentiation of spotted fever group rickettsiae by sequencing and analysis of restriction fragment length polymorphism of PCR-amplified DNA of the gene encoding the protein rOmpA. J Clin Microbiol. 1996;34 9:2058-65; doi: 10.1128/jcm.34.9.2058-2065.1996.

3. Choi YJ, Jang WJ, Kim JH, Ryu JS, Lee SH, Park KH, et al. Spotted fever group and typhus group rickettsioses in humans, South Korea. Emerg Infect Dis. 2005;11 2:237-44; doi: 10.3201/eid1102.040603.

4. Buysse M, Duron O. Two novel *Rickettsia* species of soft ticks in North Africa: *'Candidatus* Rickettsia africaseptentrionalis' and *'Candidatus* Rickettsia mauretanica'. Ticks Tick Borne Dis. 2020;11 3:101376; doi: 10.1016/j.ttbdis.2020.101376.

5. Norman AF, Regnery R, Jameson P, Greene C, Krause DC. Differentiation of *Bartonella*-like isolates at the species level by PCR-restriction fragment length polymorphism in the citrate synthase gene. J Clin Microbiol. 1995;33 7:1797-803; doi: 10.1128/jcm.33.7.1797-1803.1995.

6. Schorn S, Pfister K, Reulen H, Mahling M, Silaghi C. Occurrence of *Babesia* spp., *Rickettsia* spp. and *Bartonella* spp. in *Ixodes ricinus* in Bavarian public parks, Germany. Parasite Vector. 2011;4; doi: Artn 13510.1186/1756-3305-4-135.

7. Parola P, Roux V, Camicas JL, Baradji I, Brouqui P, Raoult D. Detection of ehrlichiae in African ticks by polymerase chain reaction. T Roy Soc Trop Med H. 2000;94 6:707-8; doi: Doi 10.1016/S0035-9203(00)90243-8.

8. Ybanez AP, Perez ZO, Gabotero SR, Yandug RT, Kotaro M, Inokuma H. First molecular detection of *Ehrlichia canis* and *Anaplasma platys* in ticks from dogs in Cebu, Philippines. Ticks Tick-Borne Dis. 2012;3 5-6:287-92; doi: 10.1016/j.ttbdis.2012.10.032.

9. Casati S, Sager H, Gern L, Piffaretti JC. Presence of potentially pathogenic *Babesia* sp for human in *Ixodes ricinus* in Switzerland. Ann Agr Env Med. 2006;13 1:65-70.

10. Bunikis J, Garpmo U, Tsao J, Berglund J, Fish D, Barbour AG. Sequence typing reveals extensive strain diversity of the Lyme borreliosis agents *Borrelia burgdorferi* in North America and *Borrelia afzelii* in Europe. Microbiol-Sgm. 2004;150:1741-55; doi: DOI 10.1099/mic.0.26944-0.
